# Microplate-based high throughput screening procedure for the isolation of lipid-rich marine microalgae

**DOI:** 10.1186/1754-6834-4-61

**Published:** 2011-12-22

**Authors:** Hugo Pereira, Luísa Barreira, André Mozes, Cláudia Florindo, Cristina Polo, Catarina V Duarte, Luísa Custódio, João Varela

**Affiliations:** 1 Centre of Marine Sciences, University of Algarve, Faro, Portugal; 2 Department of Biomedical Sciences and Medicine, University of Algarve, Faro, Portugal

**Keywords:** biofuels, BODIPY, FACS, lipid-rich strains, microalgae isolation

## Abstract

We describe a new selection method based on BODIPY (4,4-difluoro-1,3,5,7-tetramethyl-4-bora-3a,4a-diaza-*s*-indacene) staining, fluorescence activated cell sorting (FACS) and microplate-based isolation of lipid-rich microalgae from an environmental sample. Our results show that direct sorting onto solid medium upon FACS can save about 3 weeks during the scale-up process as compared with the growth of the same cultures in liquid medium. This approach enabled us to isolate a biodiverse collection of several axenic and unialgal cultures of different phyla.

## Background

Microalgae are aquatic photosynthetic microorganisms able to transform carbon dioxide into biochemicals that can later be processed into biofuels, food, feed and high-value bioactive compounds [1]. With regard to biofuels, algal biomass is considered likely to be one of the most important sources of renewable energies in the near future [2]. Although biodiesel production from microalgae is a proven technology, it still faces several technical and economical constraints that need to be addressed [3,4] in order to scale up production and thus lower the final production costs [5].

Extraction of bioactive compounds with potential applications in pharmacology and biomedicine is a relatively new trend in microalgal biotechnology. Microalgal biomass presents natural active compounds responsible for distinct biological activities, such as cytotoxic, antibiotic, antioxidant, antifungal, anti-inflammatory and antihelminthic compounds [6-9].

The rise of interest in these microscopic organisms for biotechnological applications is due to the unique biochemical features and their vast biodiversity, which to date is almost entirely unexploited [6]. Although many culture collections of microalgae have been established, the variety of unknown species and strains present in the environment with potential application in the production of biofuels and/or as a source of bioactive compounds is very high [8,10,11]. Thus, easy and feasible high throughput screening procedures are essential in order to isolate novel species and strains for specific purposes.

Although several techniques for microalgae isolation have been described previously, such as single-cell isolation in liquid and solid media, serial dilutions, medium enrichment, gravimetric separation, micromanipulation and atomized cell spray [12,13], flow cytometry has recently shown significant potential in improving microalgal strains for lipid production in an expedited fashion [14,15]. Fluorescence activated cell sorting (FACS) enables the selection of particular strains of microalgae and subsequent isolation [16]. The characterization of different populations within any mixture of cells is performed through direct measurement of optical cell properties (for example, light scatter and multicolor fluorescence emission), which in turn enables FACS of defined cell populations that can be cultured separately at a later stage [17-19].

Several authors have reported successful sorting procedures for microalgae. Reckermann [19] described the sorting and culturing of a variety of unicellular species isolated from an environmental water sample. More recently, Doan *et al. *[20] reported the isolation from Singapore waters of microalgal strains for the purpose of biofuel production. However, FACS has been considered to be a technique displaying low efficiency for the isolation of unialgal cultures, especially those of fragile species such as dinoflagellates [21]. Therefore, there is a need to develop simpler and faster methods allowing the isolation of fast-growing strains.

In the present work, a combination of two methods was tested: FACS combined with growing cells in 96-well plates containing solid agar growth medium to accelerate both the isolation procedure and culture scale-up. This combination resulted in a high throughput screening procedure to isolate and screen for lipid-rich strains by means of BODIPY 505/515 (4,4-difluoro-1,3,5,7-tetramethyl-4-bora-3a,4a-diaza-*s*-indacene) staining that can also be used to isolate fast-growing microalgae. These cells can then be further tested for bioactivities. Via this approach several strains of microalgae were isolated and easily scaled up to higher volumes at a later stage.

## Results and discussion

### Selection of fast growing strains

Algal strains intended for biotechnological applications need to be produced as fast as possible in large-scale systems in order to ensure a sustainable process. Therefore microalgae displaying high growth rates are essential. In this context, water samples were supplemented with Algal growth medium and incubated for 7 days using growth conditions as described in Methods. This enrichment step facilitates the isolation procedure, allowing fast-growing strains to dominate, by competition, other microalgae of less interest under a set of desired growth conditions. If the enrichment step is omitted, fast-growing cells might be overlooked during the isolation procedure due to their low concentration. Thus, this stage is a key step in the selection of microalgae isolated by the present method.

### Isolation by FACS

**Figure 1:**
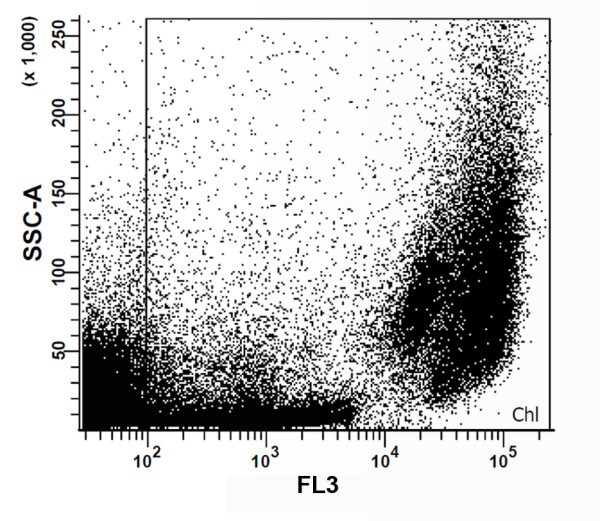
**Two-dimensional dot plot combining inner cell complexity (side scattering (SSC)) and fluorescence emission by chlorophyll (FL3)**. The Chl gate, defined as events with more than 100 arbitrary units of chlorophyll autofluorescence, corresponded to our first sorting trait in order to isolate photosynthetic cells rather than non-photosynthetic microorganisms and debris.

FACS allows simultaneous measurements of individual cells. Light scatter angles and fluorescence intensity measured in different channels allows the distinction between different clusters of cells with heterogeneous characteristics. The information given by the different channels can be visualized in two-dimensional plots combining two of the variables at each time. Figure 1 shows the result of combining side scattering (SSC) with fluorescence in the FL3 channel. The combination of these two variables enabled us to separate cells with chlorophyll from unwanted cells and debris, mainly non-photosynthetic bacteria and sediments that are normally present in natural samples. This could be accomplished as flow cytometry allows the separation of cells by the inner cell complexity and endogenous fluorescence of pigments naturally occurring in microalgae. The first property can be estimated by the measurement of side angle light scatter (SSC) [22], whereas fluorescence emitted by pigments (for example, chlorophyll, phycoerythrin and phycocyanin) can be detected in different channels upon excitation with a specific laser [19,23]. In this way, red fluorescence correlates with cellular chlorophyll content [18,19,24], which was measured in the FL3 channel. At this stage, all cells present in the dot plot displaying more than 100 arbitrary units (AU) of autofluorescence, as detected in the latter channel, were gated; this first gate was named Chl.

**Figure 2:**
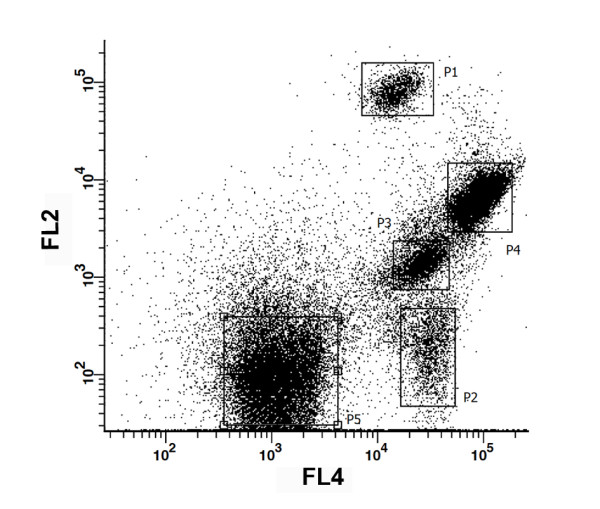
**Gating procedure performed in the dot plot combining fluorescence emission by FL2 and FL4 channels**. The boxes represent the five clusters used during the gating of an environmental sample stained with the BODIPY 505/515 (4,4-difluoro-1,3,5,7-tetramethyl-4-bora-3a,4a-diaza-*s*-indacene) solvatochromic dye.

**Figure 3:**
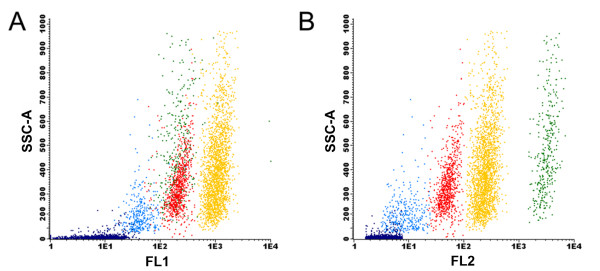
**Analysis plots combining inner cell complexity (side scattering (SSC)) with two emission channels of BODIPY (4,4-difluoro-1,3,5,7-tetramethyl-4-bora-3a,4a-diaza-*s*-indacene) stained cells**. **(A) **FL1 vs SSC. **(B) **FL2 vs SSC-A. Colors represent different gates established in Figure 2. P1 = green; P2 = light blue; P3 = red; P4 = yellow; P5 = dark blue.

**Figure 4:**
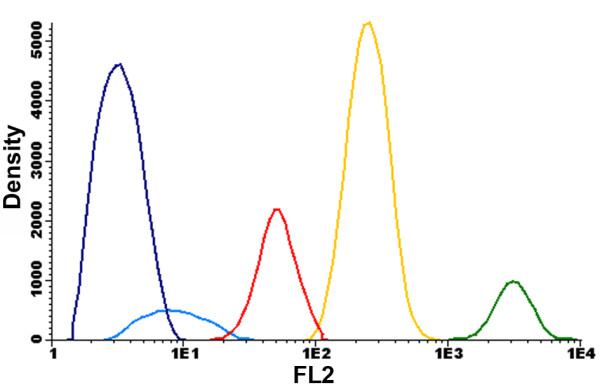
**Event density versus fluorescence in the FL2 channel**. This result shows that all gated clusters emit distinct fluorescence signals, suggesting the isolation of five different strains. Gates P4 and P5 present the highest density of events among all clusters. P1 = green; P2 = light blue; P3 = red; P4 = yellow; P5 = dark blue.

The possibility of obtaining axenic cultures is one of the main advantages of the FACS approach, since it has been shown that the latter procedure is able to remove bacteria from microalgal cultures [25]. After this initial gating, several combinations of the signal obtained with the different channels were used. From all the combined plots the best separation between populations was obtained upon combining the FL2 and FL4 channels (Figure 2). During this acquisition, well resolved clusters of cells were clearly distinguished, which simplified the gating procedure. In this way, five gates (P1, P2, P3, P4 and P5) were drawn. The signal measured in the FL4 channel is associated with phycocyanin-derived autofluorescence [19], while yellow-orange fluorescence detected in the FL2 channel is related with phycoerythrin-derived autofluorescence of cells [18,19]. In addition to measuring endogenous fluorescence, cells can also be stained with different dyes, providing a wide range of information. It has recently been shown that BODIPY is able to stain lipid bodies in photosynthetic unicellular organisms, allowing the establishment of lipid-rich microalgal cultures [26], and also the development of selection programs to improve lipid production as demonstrated with the Nile red dye [14,15]. Although BODIPY fluorescence can be detected in the FL2 channel, the highest emission intensity of this fluorochrome is in the green fluorescence bandwidth range. Hence, cell complexity (SSC) was subsequently plotted against the fluorescence emission in the FL1 channel (centered at 530/30 nm) in addition to the FL2 channel. Results obtained using these settings suggested that each cluster was a different species (Figure 3). In this figure, each gated cluster was represented by a different color in order to distinguish clusters gated in Figure 2. Interestingly, the most discriminating factor for clusters P1, P2, P3 and P4 was the fluorescence associated with the FL1 and FL2 channels (Figure 3A, B) rather than inner cell complexity (SSC) or chlorophyll fluorescence (Figure 1). The combination of BODIPY fluorescence emission with cell complexity is shown in Figure 3A. The P4 cluster displayed the highest BODIPY fluorescence and was thus selected as a good candidate source for microalgal cells with potential use for biodiesel production. P1 and P3 gates are clustered together and cannot be resolved by this combination of signals. Figure 3B combines signals from SSC and FL2 channels in which P1 and P3 clusters are resolved, as the former cluster moved to the right with respect to the P3 cluster due to a higher fluorescence emission in the FL2 channel. That may be explained by higher phycoerythrin content present in the P1 cluster cells, since the fluorescence detected in the FL1 channel was very low. Fluorescence emission detected in the FL2 channel was in fact one of the best discriminant factors for these strains. Figure 4 presents a histogram relating event (cell) density with PE fluorescence. This graph showed that well defined cell populations with distinct PE fluorescence levels could be observed and that the P4 and P5 clusters displayed the highest cell density. The P5 cluster showed significantly lower complexity and fluorescence as compared with other clusters. This low degree of complexity and fluorescence emission indicated that the cells in the latter cluster were most probably cyanobacteria [18,27].

Although BODIPY, phycoerythrin and phycocyanin fluorescence were used in the strain isolation for this specific environmental sample, different combinations of channels can be chosen to produce a similar two-dimensional plot as shown in Figure 2. In this particular case, FL2 and FL4 produced the best clustering for the chosen gating method. Though the other channels gave relevant information, they were not used in the procedure for separation of the different naturally occurring cell populations since they formed overlapped clusters. The use of fluorescence emission due to BODIPY staining in the gating procedure should be done with care since the concentration of lipids in the cells varies with the culture growth stage. Cells normally present higher lipid concentrations during stationary phase as compared with cells growing exponentially. As in the present work environmental samples underwent a pre-enrichment step to isolate fast-growing cells able to withstand competition from other microalgae, it is possible that lipid-rich strains growing actively might have been overlooked.

To assess cell morphology, the gated clusters were then sorted directly to microscope slides and observed in a Zeiss Axio Imager Z2 fluorescence microscope. Cells from the P1 cluster did not survive the cell sorting procedure, since upon microscopic observation only disrupted cells were found. The same problem was described by Reckermann [19] with *Fibrocapsa japonica *that disrupted soon after sorting. Microscopic observations suggested that the established gates were able to isolate monoalgal cultures since all observed fields from the same cluster showed the same strain. As expected, P5 was an exception and microscopic observation showed different cyanobacterial strains and debris.

**Figure 5:**
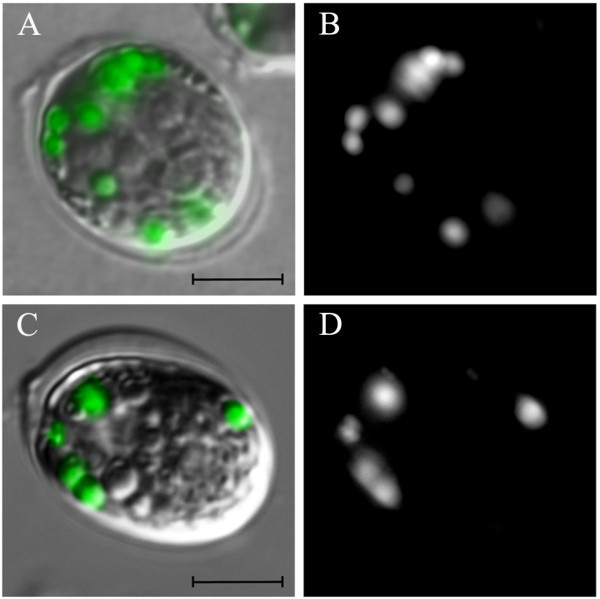
**Cells from the P4 gate internalized BODIPY (4,4-difluoro-1,3,5,7-tetramethyl-4-bora-3a,4a-diaza-*s*-indacene) in lipid bodies**. Cells were stained and sorted directly onto microscope slides after the sorting procedure. BODIPY dots could be observed with the FL1 channel **(B, D)**, and in the merged differential interference contrast (DIC) system + BODIPY fluorescence **(A, C)**. We clearly detected that BODIPY effectively stained lipid bodies in this strain (DIC in grey, BODIPY in green; scale bar = 5 μm).

Microscopic observation of cells from the P4 cluster, which presented the highest levels of fluorescence in the FL1 channel, confirmed the presence of several lipid bodies. As can be observed in Figure 5, several dots of BODIPY fluorescence were detected (Figure 5B, D), confirming that the dye was internalized into the cells, as it can be seen in the merged differential interference contrast (DIC) system plus BODIPY image (Figure 5A, C). This observation strongly supports our FACS results, suggesting that the combination of the gates used was efficient in the isolation and identification of lipid-rich microalgae that can be later tested for biodiesel production (Figure 5).

### Efficiency of cell sorting (solid medium vs liquid medium)

**Figure 6:**
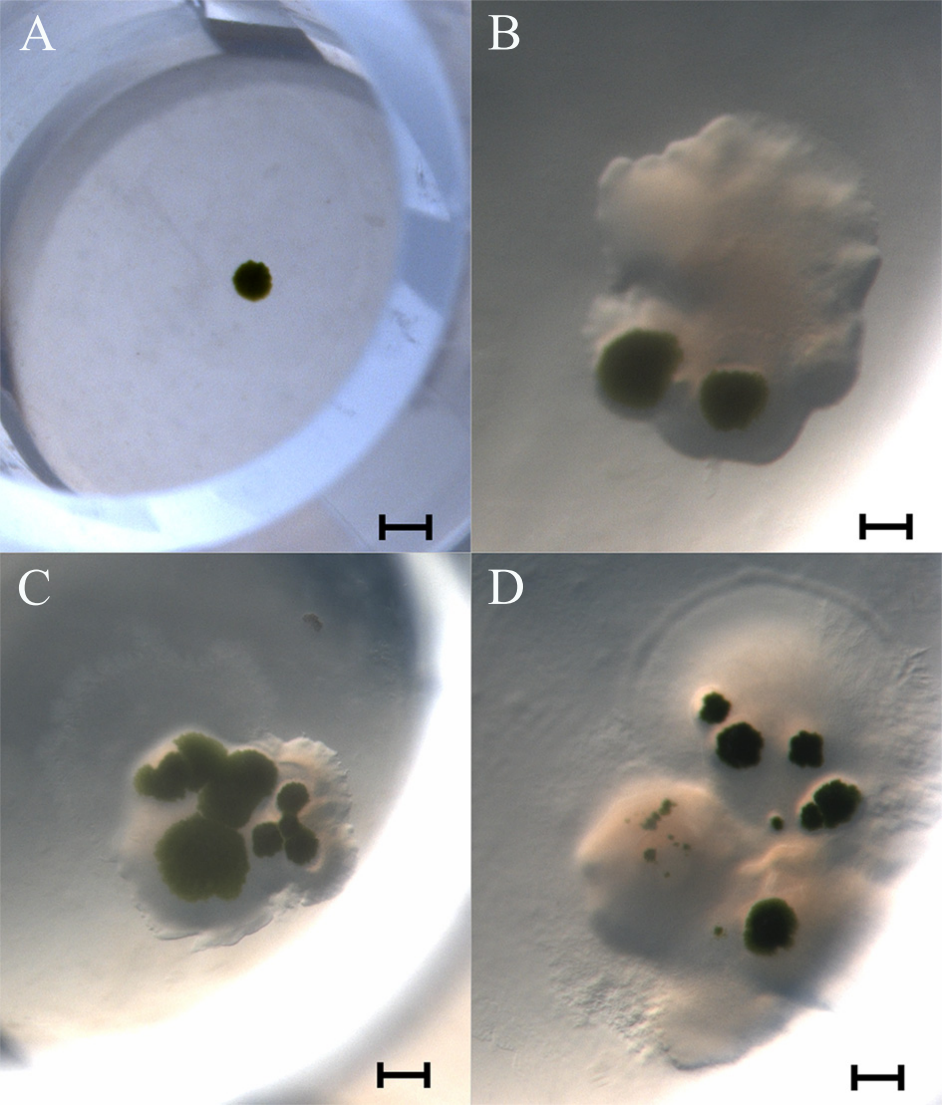
**Sorted cells were able to grow on 96-well plates containing solid agar growth medium**. After a 2-week incubation period, sorting a single event resulted in an axenic microalgal colony **(A)**. The growth of several microalgal colonies obtained from more than one sorted event is shown in panels **(B)**, **(C) **and **(D)**. However, unlike in (A), these wells contained contaminating bacteria as well (brightfield images, scale bar = 50 mm).

**Figure 7:**
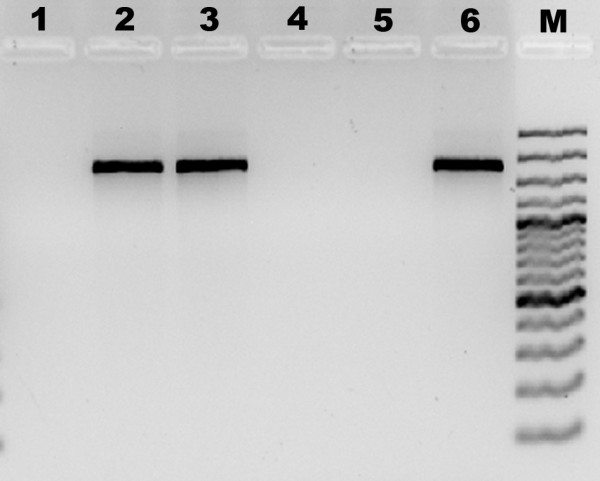
**Axenicity assessment of established culture by means of 16S rDNA PCR amplification**. The 16S rDNA was amplified with specific primers as described by Weisburg *et al. *[28]. PCR products were separated by electrophoresis in a 1.5% agarose gel and were stained with GelRed. Lane 1: negative control in which DNA was omitted. Lanes 2 and 3: PCR product derived from cultures scored visually as non-axenic cultures. Lanes 4 and 5: PCR reaction from DNA samples of cultures scored visually as axenic cultures. Lane 6: positive control (*Vibrio shiloi *DNA sample). Lane M: molecular weight marker (Gene Ruler 100 bp Plus DNA ladder, 100 to 3,000 bp; Fermentas GmbH, St. Leon-Rot, Germany).

Growth was visible in solid medium in approximately 70% of the wells after 2 weeks of incubation (Figure 6), whereas 20% did not show any sign of bacterial growth (Figure 6A). Absence or presence of bacteria was later confirmed by means of PCR amplification of bacterial 16S ribosomal DNA (Figure 7) using primers as described by Weisburg *et al. *[28]. In liquid medium, however, after 2 weeks only 45% of wells presented algal growth and almost 95% of these displayed visible bacterial growth. However, in this medium species that did not grow on solid medium were successfully isolated. Cells from the P3 cluster did not grow on solid growth medium, although with some difficulty these microalgae were able to proliferate in liquid medium. In fact, some strains and even entire algal taxa (for example, dinoflagellates) hardly grow on agar growth medium [12]. However, isolation in solid medium showed obvious advantages, such as (1) faster biomass growth at the early stages of the scale-up process; (2) easy application and maintenance of cultures, since in liquid medium microalgae need to be kept afloat to promote growth efficiently; and (3) bacterial growth is detected more easily.

The efficiency of the sorting procedure obtained in this work (70%) was considerably higher than that reported by Sensen *et al. *[25] for the removal of bacteria from unialgal cultures (20% to 30%). Global recovery, however, was lower than reported by Doan *et al. *[20] in novel isolates from an environmental water sample (82% to 100%). Still, sorting efficiency is highly dependent on the original sample, including the starting group of species and their abundance, thus demonstrating the importance of the initial enrichment step. For instance, Sinigalliano *et al. *[21] compared the isolation efficiency of dinoflagellates between FACS and manual picking using a micropipette and the former approach appeared to be less efficient than the latter (0.5% vs 2%, respectively). This could be due to the frailness of algae belonging to this taxonomic group. The enrichment step can also lead to a selective enrichment of some species. In our work, Algal growth medium, which contains low levels of silica, was used [29]. Therefore, domination of mixed cultures by diatoms and other silicate-requiring algae was not favored during the enrichment process.

To ensure algal growth and estimate the best starting number of cells for an expedited recovery, different numbers of events (cell-containing droplets) were tested. For each well 1, 2, 10 or 20 events were sorted. Interestingly, the starting number of cells per well was not a constraint in the scale-up procedure on solid medium. Regardless of the number or events sorted per well, visual growth was obtained after a 2-week incubation period. Single cell sorting in solid medium (one event) yielded a single axenic colony in most wells (Figure 6A), which was easily transferred onto a Petri dish with solid medium. Wells with > 1 event (2, 10, 20 events) gave rise to a large number of colonies; however, all of them presented visible bacterial growth (Figure 6B-D).

In liquid medium, after a 2-week incubation period algal growth was only visible in the wells with 10 or 20 events. Most of the obtained cultures were not unialgal and showed abundant bacterial contamination as verified by microscopic observation (data not shown). Algal growth in wells with one or two events was only visible after 4 weeks. A few of these cultures were, however, axenic, though at a very low frequency (5%). Therefore, in liquid medium, increased culture growth can be achieved by sorting more events, which results in a faster scale-up, but the use of several replicates is recommended when sorting to liquid medium to isolate axenic cultures as this improves the scale-up procedure efficiency. Overall, the scale-up procedure was considerably faster in solid medium (4 weeks) than the required period for achieving the same growth and culture volume using liquid medium (7 weeks).

The fact that BODIPY cell staining did not affect cell recovery in both solid and liquid medium is essential for present and future work in the detection and isolation of lipid hyper-producing algae. Although it has already been reported that culturing cells after Nile red staining is possible [14,15], BODIPY presents several advantages when compared with Nile red: (1) BODIPY is able to stain a wide range of algae groups without the need of using high concentrations of dimethylsulfoxide (DMSO) or acetone to carry the dye in, which can be key to wider and faster cell recovery and scale-up; and (2) BODIPY preferentially traces lipid bodies instead of other cytoplasmic compartments [26].

### Identification of microalgae

Although light and electron microscopy have been traditionally used to identify microalgae, small subunit ribosomal RNA gene (16S or 18S rDNA) sequences are considered as an ideal tool in the identification of microorganisms, including microalgae [30-32]. Thus, after the scale-up procedure, isolated strains were identified by microscopy and amplification with specific primers by PCR of the 18S rDNA sequences. All cyanobacteria were kindly identified by the microbiology company AquaExam by means of microscopy. Isolates belong to three different phyla (Table 1). Strains P4 and P2 are chlorophytes, *Tetraselmis *sp. and *Nannochlorum *sp., respectively. The P3 gate corresponded to an unclassified chrysophyte. From the P5 gate one cyanobacterial strain of the genus *Synechococcus *was successfully isolated. The monoalgal status of the cultures was ensured by repeated subculturing from isolated colonies followed by 18S rDNA sequencing, which yielded sequences 100% identical when comparing different colonies of a given strain.

**Table 1:** Strains isolated during the sorting procedure

Cluster	Strain	Phylum
P1	Unidentified	
P2	*Nannochlorum *sp.	Chlorophyta
P3	Unclassified chrysophyte	Ochrophyta
P4	*Tetraselmis *sp.	Chlorophyta
P5	*Synechococcus *sp.	Cyanobacteria

Upon its optimization, this method, coupling fluorescence activated cell sorting with BODIPY staining, was efficiently applied to several water samples collected in different marine habitats, namely salt marsh inlets (Ria Formosa, Almargem) and former salt ponds near the estuary of the Guadiana river used nowadays for fish farming (Atlantik Fish), which resulted in the isolation of 56 strains. From these, 12 fast-growing strains were selected, isolated and successfully scaled up to higher volumes (Table 2). The majority of strains isolated by this method belong to the phylum Chlorophyta (approximately 70%), *Nannochloris *and *Tetraselmis *being the most abundant genera. During this work, only 10% of the isolated strains were diatoms due to the unfavorable conditions of the initial enrichment step of the water samples. Accordingly, only two diatom strains (phylum Heterokontophyta) were successfully scaled up: *Nitzschia *sp. and *Cyclotella *sp. Cyanobacteria was the second most abundant phylum isolated by this method, representing 20% of the total isolates and included *Pseudanabaena *sp., *Leptolyngbya *sp. and *Synechococcus *sp. In order to isolate more cyanobacterial strains a different gating method will be needed.

**Table 2:** Species and strains isolated throughout this work, corresponding phylum and methods applied in the identification of each strain

Strain	Phylum	Identification method
*Pseudanabaena *sp.	Cyanobacteria	Microscopy: AquaExam
*Synechococcus *sp.	Cyanobacteria	Microscopy: AquaExam
*Leptolyngbya *sp.	Cyanobacteria	Microscopy: AquaExam
*Chlorella *sp.	Chlorophyta	18S rDNA sequencing
*Tetraselmis *sp.	Chlorophyta	18S rDNA sequencing
*Tetraselmis *sp.	Chlorophyta	18S rDNA sequencing
*Tetraselmis *sp.	Chlorophyta	18S rDNA sequencing
*Nannochlorum *sp.	Chlorophyta	18S rDNA sequencing
*Nannochlorum *sp.	Chlorophyta	18S rDNA sequencing
Unclassified chrysophyte	Ochrophyta	18S rDNA sequencing
*Cyclotella *sp.	Heterokontophyta	18S rDNA sequencing
*Nitzschia *sp.	Heterokontophyta	18S rDNA sequencing

### Growth and lipid assessment

**Figure 8:**
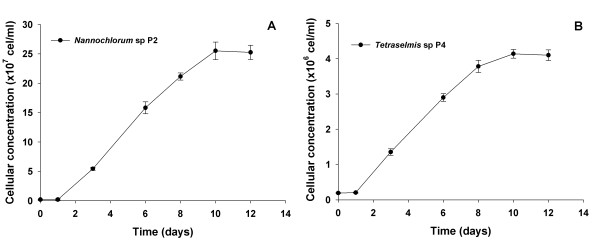
**Growth curve of established isolates with potential application as lipid sources**. *Nannochlorum *sp. P2 **(A) **and *Tetraselmis *sp. P4 **(B) **were able to grow exponentially and reach stationary phase in approximately 10 days. Cellular concentration was obtained upon cell counting in a Neubauer chamber (*n *= 6).

In order to confirm the assessment of lipid-rich microalgae by BODIPY staining (Figure 3A, B), the growth curve and total lipid contents of the *Nannochlorum *sp. P2 and *Tetraselmis *sp. P4 strains were determined (Figure 8). Both strains presented high growth rate, reaching the exponential growth stage in approximately 10 days and cell concentrations of about 2.5 × 10^8 ^(P2) and 4.0 × 10^6 ^cells/ml (P4) in the stationary phase. The disparity in number of cells at the stationary phase between the two strains is most probably due to low cell size of *Nannochlorum *sp. P2 (approximately 2 μm) when compared to *Tetraselmis *sp. (approximately 9 μm). Total lipid concentration was significantly higher (*P *< 0.01) in *Tetraselmis *sp. P4 (25.4% of dry weight) as compared to *Nannochlorum *sp. P2 (18.1% of the dry weight). These results confirm that cells derived from the P4 cluster accumulate higher amounts of lipids as anticipated by the fluorescence measurements obtained during FACS.

## Conclusions

Flow cytometry coupled with fluorescence activated cell sorting is an efficient tool for isolating strains of microalgae, which can be used either as biodiesel raw material, or as a source for bioactive compounds. Our FACS approach is a user-friendly, fast procedure than most common methods for the isolation of microalgae, with the advantage of being able to obtain axenic, unialgal cultures in a matter of weeks. This resulted in a 4-week culture scale-up (instead of a 7-week scale-up) if cells were sorted directly onto solid rather than liquid medium.

A wide range of microalgae groups has already been isolated by this method. This suggests that the method described here is a promising high throughput procedure to isolate lipid-rich strains as well as microalgae for other biotechnological purposes. The gating procedure and culture medium added in the initial enrichment step are key to favor the isolation of fast-growing microalgal taxa with high lipid contents.

## Methods

### Sampling and microalgal growth

Water sampling was performed in aquaculture ponds at the facilities of Atlantik Fish SA on the south-eastern coast of Portugal. In every sampling spot the water was collected and stored in 1 l bottles and kept at room temperature. At a later stage, the water samples were transferred to 80 ml test tubes containing liquid Algal growth medium, which was prepared with sterile seawater supplemented with an Algal stock solution concentrated 1,000 × [29]. Cultures were kept in an incubator for 7 days at 21°C, with a 12:12 h dark/light photoperiod, at a photon flux density of 80 μmol/m^2^/s. Growth on solid medium was carried out in either Petri dishes or 96-well plates containing Algal growth medium solidified with 1.5% agar.

### Cell staining

Lipid bodies were stained with a solvatochromic fluorochrome, BODIPY 505/515 (Life Technologies Europe BV, Porto, Portugal), as described by Cooper *et al. *[26]. Cells were stained with a 1 mM aqueous solution of BODIPY dissolved in DMSO (0.2%) to attain a final concentration of 1 μM. Upon addition of the fluorochrome, tubes were vortexed for 1 minute and incubated at room temperature in darkness for 10 minutes.

### FACS

The flow cytometer used in our studies was a Becton Dickinson FACS Aria II (BD Biosciences, Erembodegem, Belgium). Fluorescence readings were performed by excitation with a blue and red laser (488 and 633 nm, respectively). The emission signal was measured in three channels upon excitation with the blue laser: FL1 channel centered at 530/30 nm; FL2 centered at 585/42 nm; and FL3 centered at 695/40 nm. A fourth channel, FL4, registered the emission at 660/20 nm after excitation with the red laser.

Samples were acquired with the software FACSDiva version 6.1.3 (BD Biosciences, Erembodegem, Belgium). After the acquisition of samples, images were treated with the analysis software, Infinicyt 1.5.0 (Cytognos S.L., Santa Marta de Tormes, Spain).

The settings and compensations of all channels and lasers were the same for all sorting procedures. The flow cytometry sheath fluid used in all experiments was sterile filtered seawater. Filters (PALL) used had a pore size of 0.2 μm. Sorting was performed at 2,000 events/s flow rate using 'single cell' sort precision mode, with a 100 μm nozzle.

Cells were sorted directly into wells of 96-well plates containing 250 μl of either liquid or solid (agar) Algal growth medium. In order to assess the best number of cells needed to achieve visible culture growth in a feasible time, sorting conditions were set in order to direct 1, 2, 10 or 20 droplets into each well. After the cell sorting procedure, cells were incubated for 2 weeks in the same incubator and growth conditions as described above.

### Microscopy

Microscopic images were acquired in a Zeiss AXIOMAGER Z2 microscope, with a coollSNApHQ2 camera and AxioVision software version 4.8 (Carl Zeiss MicroImaging GmbH, Gõttingen, Germany), using the 100 × lens. For the fluorescence images, we used Zeiss 38 He filter set (Carl Zeiss MicroImaging GmbH, Gõttingen, Germany) for fluorescein isothiocyanate (FITC) and the transmitted light images were acquired using differential interference contrast. Z stacks were acquired and the resulting image was a maximum intensity projection of the stacks. For the plate cultures the images where acquired in a Zeiss SteREO Lumar.V12 stereoscope, equipped with an Axiocam MRC, using AxioVision software release 4.8 (Carl Zeiss MicroImaging GmbH, Gõttingen, Germany). Images were treated using Image J software (Research Service Branch, NIH, Bethesda, MD).

### Culture scale-up

The scale-up procedure was carried out by streaking a single colony from a well that did not show any signs of bacterial growth onto a Petri dish containing solid Algal growth medium. After 1 week axenic plates were scrapped to 100 ml Erlenmeyer flasks with liquid Algal growth medium. A week later the 100 ml culture was transferred to 1 l reactors with aeration. In liquid medium, the scale-up was performed by transferring the 250 μl of the 96-well plates into 5 ml test tubes containing 1 ml of liquid Algal growth medium under minor aeration. Cells were allowed to grow for 1 week and were then transferred successively to 20 ml test tubes, 100 ml test tubes and finally 1 l reactors with aeration.

### Gravimetric determination of total lipids

Lipid extraction was performed according to a modified protocol by Bligh and Dyer [33]. Briefly, the obtained algal biomass was homogenized at room temperature with an IKA Ultra-Turrax disperser (IKA-Werke GmbH, Staufen, Germany), in a mixture of chloroform, methanol and water (2:2:1). The mixture was centrifuged to allow phase separation, and a known volume of the organic phase was pipetted into a new preweighed tube. The extract was then evaporated until dryness in a warm bath (50°C) and weighed carefully to estimate lipid contents.

### Taxonomic identification

The resulting unialgal cultures were identified by optical microscopy and 18S rDNA sequencing. DNA extraction was performed with the EZNA DNA plant extraction kit (Omega Bio-Tek, Norcross, GA) according to the manufacturer's procedure. The obtained DNA was amplified by PCR with specific primers (Table 3) and sequenced at an in-house DNA sequencing facility equipped with an Applied Biosystems 3130XL DNA sequencer (Life Technologies BV, Porto, Portugal).

**Table 3:** Primers used in the identification of the isolated strains (18S rDNA) and determination of axenicity status of established microalgal cultures (16S rDNA)

Gene	Primer	Sequence (5' to 3')
18S	18SUnivFor	ACCTGGTTGATCCTGCCAGT
18S	18SUnivRev	TCAGCCTTGCGACCATAC
16S	F27	AGAGTTTGATCMTGGCTCAG
16S	R1492	TACGGYTACCTTGTTACGACTT

## Competing interests

Funding for this and past works carried out at the MarBiotech group (the name of the research group the authors belong to) of the Center of Marine Sciences (CCMAR) was obtained from public sources only. Thus, the MarBiotech research team does not belong or is not otherwise financially linked to a company that may lose or gain from these results. The manuscript processing charge will also be paid by public funds, namely by CCMAR's institutional budget allocated to the MarBiotech research group. None of the authors holds stocks or shares in an organization that may in any way gain or lose financially with the publication of this manuscript, nor have the authors tried to secure a patent pertaining the contents of the manuscript.

## Authors' contributions

HP and CP performed the environmental sampling and algal growth under the supervision of LC and JV. Flow cytometry and data analysis thereof were carried out by AM, HP and CP under the supervision of LB and JV. All microscopy work, image processing and analyses were performed by CF, HP and CVD under the supervision of LB and JV. All authors read and approved the final manuscript.

## Authors' information

The MarBiotech research group belongs to CCMAR http://www.ccmar.ualg.pt, an institution dedicated to carry out research in marine sciences belonging to ASSEMBLE (the Association of European Marine Biological Laboratories; http://www.assemblemarine.org; CCMAR is named as 'Faro'). MarBiotech is led by JV, who has published several works in the elucidation of the biosynthesis pathway of lipophyllic pigments (carotenoids) in the microalga *Dunaliella salina*. Recently JV has secured the collaboration of one postdoctoral fellow (LC) and a faculty member (LB), who provided funding and expertise concerning analytical methods for lipid detection. HP and CVD are recipients of research grants for graduate students. AM and CF are members of the University of Algarve (where CCMAR is located) and are technical staff specialized in flow cytometry and microscopy, respectively.

## References

[B1] Walker TL Purton S Becker DK Collet C Microalgae as bioreactors Plant Cell Rep 2005 24 629 641 10.1007/s00299-005-0004-6 16136314

[B2] Demirbas A Use of algae as biofuel sources Energ Convers Manage 2010 51 2738 2749 10.1016/j.enconman.2010.06.010

[B3] Pienkos P Darzins A The promise and challenges of microalgal-derived biofuels Biofuel Bioprod Bior 2009 3 431 440 10.1002/bbb.159

[B4] Oltra C Stakeholder perceptions of biofuels from microalgae Energ Policy 2011 39 1774 1781 10.1016/j.enpol.2011.01.009

[B5] Wijffels RH Barbosa MJ An outlook on microalgal biofuels Science 2010 329 796 799 10.1126/science.1189003 20705853

[B6] Pulz O Gross W Valuable products from biotechnology of microalgae Appl Microbiol Biotechnol 2004 65 635 648 10.1007/s00253-004-1647-x 15300417

[B7] Gouveia L Batista AP Sousa I Raymundo A Bandarra NM Papadopoulos KN Microalgae in novel food products Food Chemistry Research Developments 2008 Hauppauge, NY: Nova Science Publishers, Inc. 1 37

[B8] Plaza M Herrero M Cifuentes A Ibáñez E Innovative natural functional ingredients from microalgae J Agric Food Chem 2009 57 7159 7170 10.1021/jf901070g 19650628

[B9] Patil KJ Patil VA Mahajan SR Mahajan RT Bio-activity of algae belonging to Bhusawal region, Maharashtra Curr Bot 2011 2 29 31

[B10] Ördög V Stirk WA Lenobel R Bancírová M Strnad M van Staden J Szigeti J Németh L Screening microalgae for some potentially useful agricultural and pharmaceutical secondary metabolites J App Phycol 2004 16 309 314

[B11] Mata TM Martins AA Caetano NS Microalgae for biodiesel production and other applications: a review Renew Sust Energ Rev 2010 14 217 232 10.1016/j.rser.2009.07.020

[B12] Andersen RA Kawachi M Andersen RA Traditional microalgae isolation techniques Algal Culturing Techniques 2005 New York, NY: Academic Press 83 100

[B13] Mutanda T Ramesh D Karthikeyan S Kumari S Anandraj A Bux F Bioprospecting for hyper-lipid producing microalgal strains for sustainable biofuel production Bioresource Technol 2011 102 57 70 10.1016/j.biortech.2010.06.077 20624676

[B14] Doan TY Obbard JP Enhanced lipid production in *Nannochloropsis *sp. using fluorescence-activated cell sorting GCB Bioenergy 2010 3 264 270

[B15] Montero MF Aristizábal M Reina GG Isolation of high-lipid content strains of the marine microalga *Tetraselmis suecica *for biodiesel production by flow cytometry and single-cell sorting J Appl Phycol 2010 23 1053 1057

[B16] Acreman J Algae and cyanobacteria: isolation, culture and long-term maintenance J Ind Microbiol 1994 13 193 194 10.1007/BF01584008

[B17] Hofstraat JW de Vreeze MEJ van Zeijll WJM Peperzak L Peeters JCH Balvoort HW Flow cytometric discrimination of phytoplankton size classes by fluorescence emission and excitation properties J Fluoresc 1991 1 249 265 10.1007/BF00865249 24243075

[B18] Veldhuis MJW Kraay GW Application of flow cytometry in marine phytoplankton research: current applications and future perspectives Sci Mar 2000 64 121 134

[B19] Reckermann M Flow sorting in aquatic ecology Sci Mar 2000 64 235 246

[B20] Doan TY Sivaloganathan B Obbard JP Screening of marine microalgae for biodiesel feedstock Biomass Bioenerg 35 2534 2544

[B21] Sinigalliano CD Winshell J Guerrero MA Scorzetti G Fell JW Eaton RW Brand L Rein KS Viable cell sorting of dinoflagellates by multiparametric flow cytometry Phycologia 2009 48 249 257 10.2216/08-51.1 20305733 PMC2841404

[B22] Nunez R Flow cytometry: principles and instrumentation Curr Issues Mol Biol 2001 3 39 45 11471975

[B23] Dubelaar GBJ Jonker RR Flow cytometry as a tool for the study of phytoplankton Sci Mar 2000 64 135 156

[B24] Jochem FJ Probing the physiological state of phytoplankton at the single-cell level Sci Mar 2000 64 183 195

[B25] Sensen CW Heimann K Melkonian M The production of clonal and axenic cultures of microalgae using fluorescence-activated cell sorting Eur J Phycol 1993 28 93 97 10.1080/09670269300650151

[B26] Cooper MS Hardin WR Petersen TW Cattolico RA Visualizing "green oil" in live algal cells J Biosci Bioeng 2010 109 198 201 10.1016/j.jbiosc.2009.08.004 20129108

[B27] Marie D Simon N Vaulot D Andersen RA Phytoplankton cell counting by flow cytometry Algal Culturing Techniques 2005 New York, NY: Academic Press 253 268

[B28] Weisburg WG Barns SM Lane DJ 16S ribosomal DNA amplification for phylogenetic study J Bacteriol 1991 173 697 703 1987160 10.1128/jb.173.2.697-703.1991 PMC207061

[B29] Fábregas J Abalde J Herrero C Cabezas BV Veiga M Growth of the marine microalga *Tetraselmis suecica *in batch cultures with different salinities and nutrient concentrations Aquaculture 1984 42 207 215 10.1016/0044-8486(84)90101-7

[B30] Olsen GJ Lane DJ Giovannoni SJ Pace NR Microbial ecology and evolution: a ribosomal RNA approach Annu Rev Microbiol 1986 40 337 365 10.1146/annurev.mi.40.100186.002005 2430518

[B31] Jayarao BM Doré JE Baumbach GA Matthews KR Oliver SP Differentiation of *Streptococcus uberis *from *Streptococcus parauberis *by polymerase chain reaction and restriction fragment length polymorphism analysis of 16S ribosomal DNA J Clin Microbiol 1991 29 2774 2778 1684585 10.1128/jcm.29.12.2774-2778.1991 PMC270431

[B32] Olmos J Paniagua J Contreras R Molecular identification of *Dunaliella *sp utilizing the 18S rDNA gene Lett App Microbiol 2000 30 80 84 10.1046/j.1472-765x.2000.00672.x 10728567

[B33] Bligh EG Dyer WJ A rapid method for the total lipid extraction and purification Can J Biochem Physiol 1959 37 911 917 10.1139/o59-099 13671378

